# Supplementary material for: Magnetic sustentation as an adsorption characterization technique for paramagnetic metal-organic frameworks
Source: Commun Chem. 2023 Jan 5;6:4. doi: 10.1038/s42004-022-00799-w (PMC9814357; doi:10.1038/s42004-022-00799-w)
Supplement: Supplementary file 2 — Supplementary Information [file 42004_2022_799_MOESM2_ESM.pdf]

Supporting information

MAGNETIC SUSTENTATION AS AN ADSORPTION CHARACTERIZATION TECHNIQUE FOR PARAMAGNETIC METAL-ORGANIC FRAMEWORKS

Nagore Barroso[a], Jacopo Andreo[a], Garikoitz Beobide[a,b], Oscar Castillo[a,b,*], Antonio Luque[a,b], Sonia Pérez-Yáñez[a,b] and Stefan Wuttke[a,c,*]

[a]*BCMaterials, Basque Center for Materials, Applications and Nanostructures, UPV/EHU Science Park, 48950 Leioa, Spain*

[b]*Organic and Inorganic Chemistry Department, University of the Basque Country, UPV/EHU, Barrio Sarriena s/n, 48950 Leioa, Spain*

[c]*IKERBASQUE, Basque Foundation for Science, 48009 Bilbao, Spain*

**Correspondence: oscar.castillo@ehu.eus (O.C.), stefan.wuttke@bcmaterials.net (S.W.)*

Figures

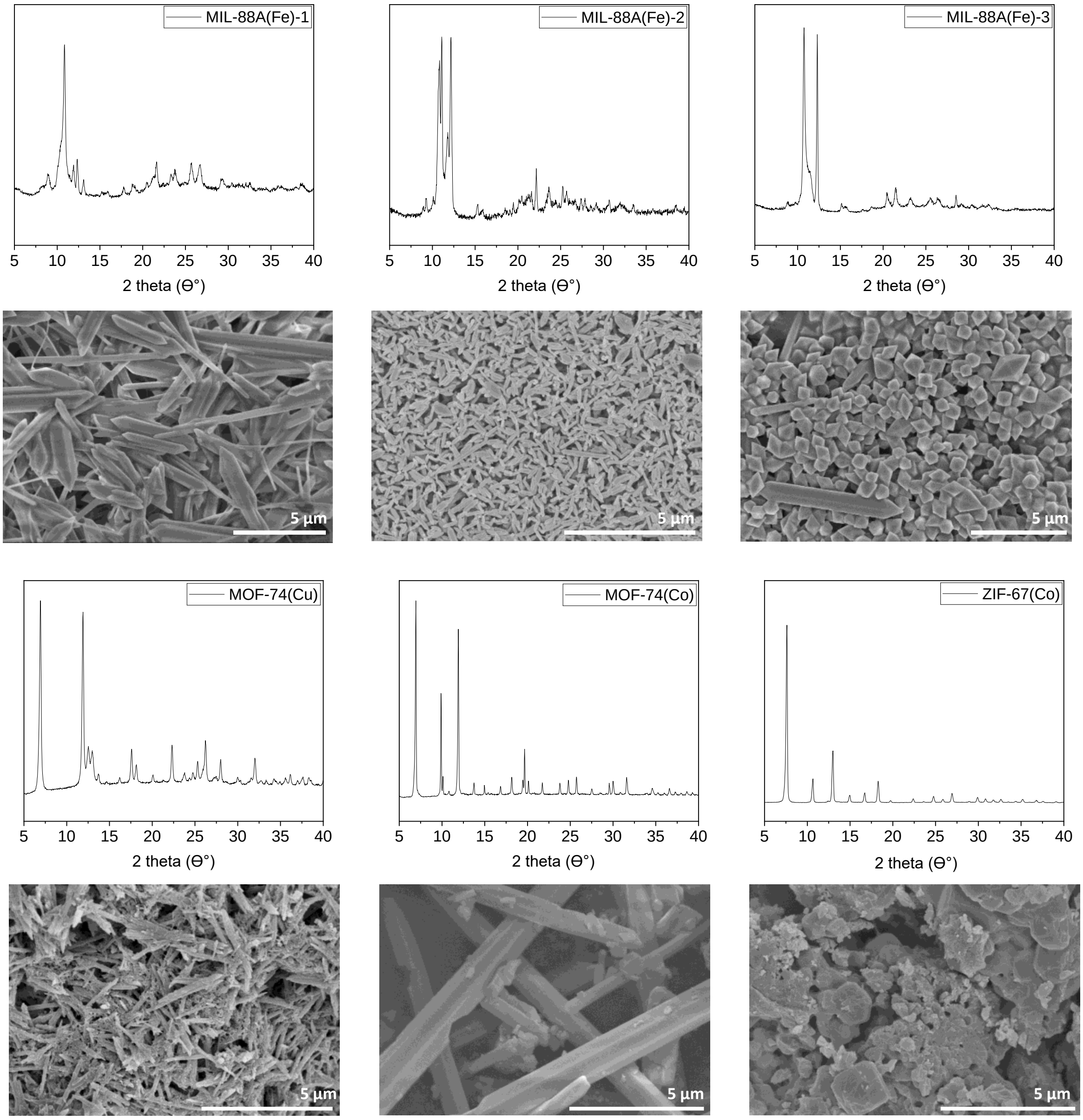

Fig. S1 Characterization of the as-synthesized MOFs. Power X-Ray Diffraction (PXRD) and scanning electron microscopy (SEM) of as-synthesized MOFs: MIL-88A(Fe)-1, 2, 3, MOF-74(Cu), MOF-74(Co) and ZIF-67(Co).

Table S1 Detailed data for MIL-88A(Fe) before and after adsorption. Critical magnetic field H(T) for as-synthesized MIL-88A(Fe) before and after adsorption, the average value and deviation.

Compound	H(T)	H(T)	H(T)	H(T)	H(T)	$H_{average}$(T)	σ(T)
MIL-88A(Fe)(Ø)	0.14963	0.14108	0.14917	0.14557	0.14287	0.14566	0.00377
Acetonitrile	0.19172	0.19172	0.18787	0.18826	0.18484	0.18888	0.00291
DMSO	0.18092	0.18396	0.18268	0.18224	0.18870	0.18370	0.00300
DMF	0.16208	0.15897	0.16208	0.16737	0.16737	0.16358	0.00369
THF	0.16208	0.15811	0.16069	0.15811	0.16428	0.16065	0.00265
Aniline	0.15408	0.15141	0.15454	0.15408	0.15277	0.15338	0.00129
Acetylsalicylic acid	0.15363	0.14785	0.15856	0.15721	0.14963	0.15338	0.00464
4-aminosalicylic acid	0.14432	0.15054	0.14917	0.15054	0.15231	0.14938	0.00304
Naproxen sodium	0.14648	0.15454	0.15054	0.14963	0.15363	0.15096	0.00324

Table S2 Detailed data for MOF-74(Cu) before and after adsorption. Critical magnetic field H(T) for as-synthesized MOF-74(Cu) before and after adsorption, the average value and deviation.

Compound	H(T)	H(T)	H(T)	H(T)	H(T)	$H_{average}$(T)	σ(T)
MOF-74(Cu)(Ø)	0.15721	0.18440	0.18484	0.19215	0.19515	0.18275	0.01501
Acetonitrile	0.31375	0.31483	0.32290	0.32179	0.29959	0.31457	0.00931
DMSO	0.28441	0.31633	0.27861	0.29733	0.28632	0.29260	0.01490
Tetramethylurea	0.25346	0.25305	0.23594	0.23974	0.23854	0.24415	0.00843
THF	0.23364	0.23240	0.25143	0.26259	0.25464	0.24694	0.01335
Aniline	0.23035	0.22663	0.25784	0.25824	0.24217	0.24305	0.01484
Acetylsalicylic acid	0.21794	0.22166	0.23444	0.23323	0.22747	0.22695	0.00716
4-aminosalicylic acid	0.22415	0.22625	0.22166	0.22830	0.22625	0.22532	0.00252

Table S3 Detailed data for MOF-74(Co) before and after adsorption. Critical magnetic field H(T) for as-synthesized MOF-74(Co) before and after adsorption, the average value and deviation.

**Compound**	**H(T)**	**H(T)**	**H(T)**	**H(T)**	**H(T)**	**$H_{average}$(T)**	**σ(T)**
**MOF-74(Co)(Ø)**	0.08243	0.08164	0.07714	0.08115	0.07829	0.08013	0.00229
**Acetonitrile**	0.10710	0.10383	0.10620	0.10478	0.11037	0.10645	0.00253
**DMSO**	0.10486	0.11084	0.10383	0.10990	0.11084	0.10805	0.00343
**Tetramethylurea**	0.09019	0.08704	0.09729	0.09394	0.09681	0.09305	0.00439
**THF**	0.10383	0.10244	0.09820	0.10054	0.09963	0.10093	0.00223
**Aniline**	0.09067	0.09224	0.09398	0.09019	0.09585	0.09259	0.00235
**Acetylsalicylic acid**	0.08212	0.08686	0.08673	0.07974	0.07926	0.08294	0.00368
**4-aminosalicylic acid**	0.08115	0.08260	0.08686	0.08309	0.08212	0.08317	0.00219

Table S4 Detailed data for ZIF-67(Co) before and after adsorption. Critical magnetic field H(T) for as-synthesized ZIF-67(Co) before and after adsorption, the average value and deviation.

**Compound**	**H(T)**	**H(T)**	**H(T)**	**H(T)**	**H(T)**	**$H_{average}$(T)**	**σ(T)**
ZIF-67(Co)(Ø)	0.09820	0.09633	0.10383	0.09868	0.10383	0.10017	0.00345
**Acetonitrile**	0.10244	0.10383	0.09868	0.10102	0.10663	0.10252	0.00298
**DMSO**	0.10292	0.10852	0.10899	0.10244	0.10383	0.10534	0.00316
**THF**	0.10054	0.11037	0.10990	0.10383	0.11037	0.10700	0.00455
**Aniline**	0.10620	0.10710	0.10102	0.10292	0.10852	0.10515	0.00310

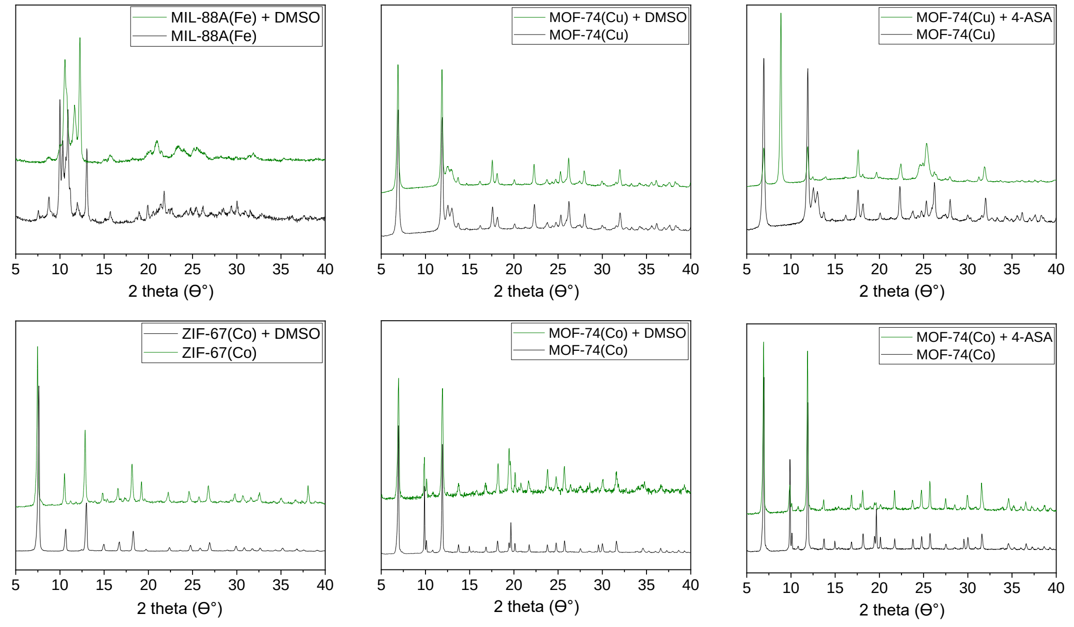

Fig. S2 PXRD after adsorption experiments. PXRD patterns of MOFs after 24 h adsorption experiment in DMSO and 4-aminoterephthalic acid.

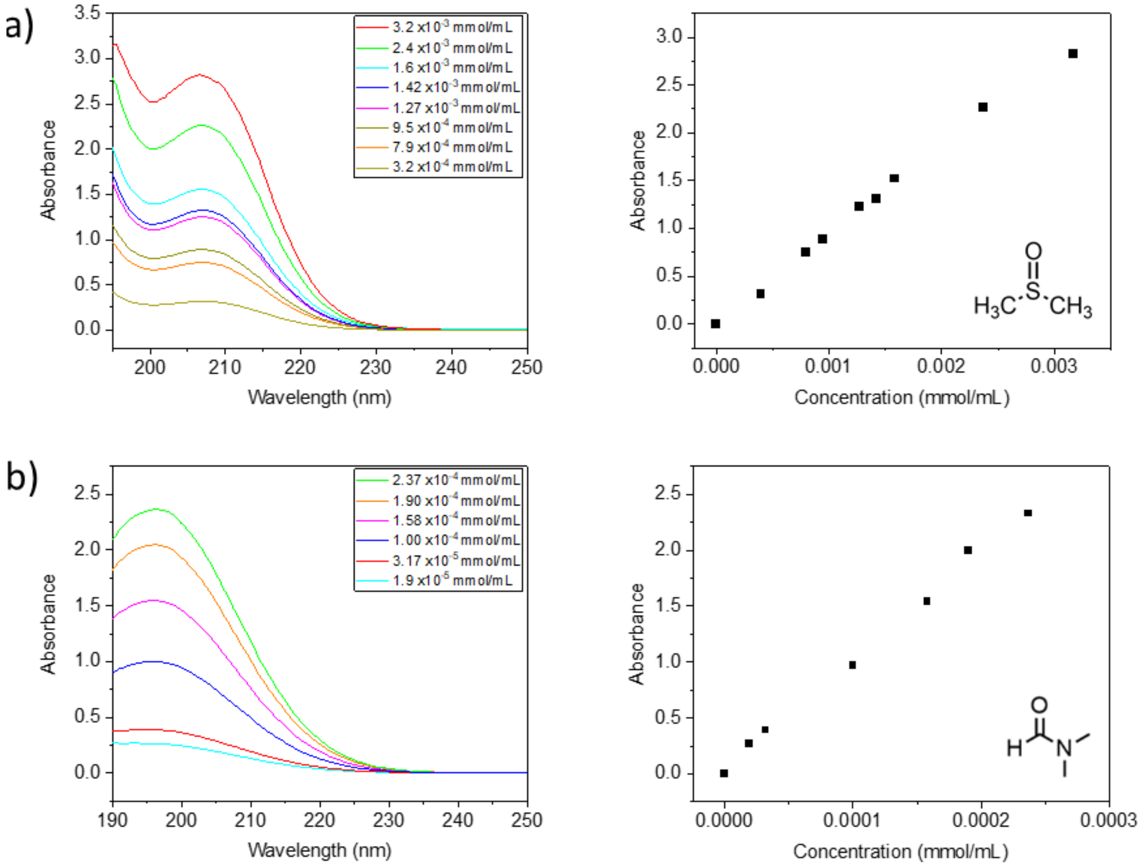

Fig. S3 Regression lines in water calculated with UV-VIS spectroscopy. Ultraviolet spectra and regression lines for a) DMSO (y = 393.21x – 57.021; R^2 = 0.997) and b) DMF (y = 9796.5x + 0.0412; R^2 = 0.996) in water.

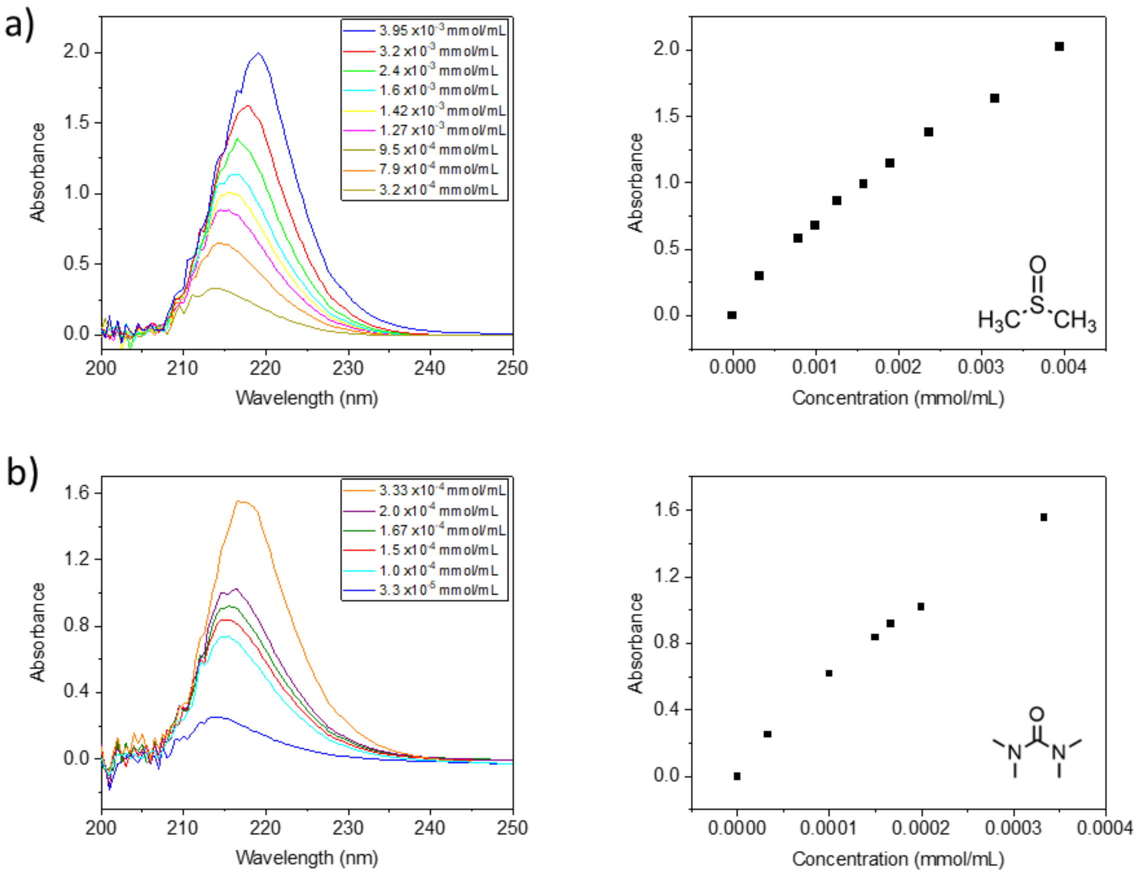

Fig. S4 Regression lines in ethanol calculated with UV-VIS spectroscopy. Ultraviolet spectra and regression lines for a) DMSO (y = 488.63x + 0.1602; R^2 = 0.984) and b) TMU (y = 4578.1x + 0.099; R^2 = 0.985) in ethanol.

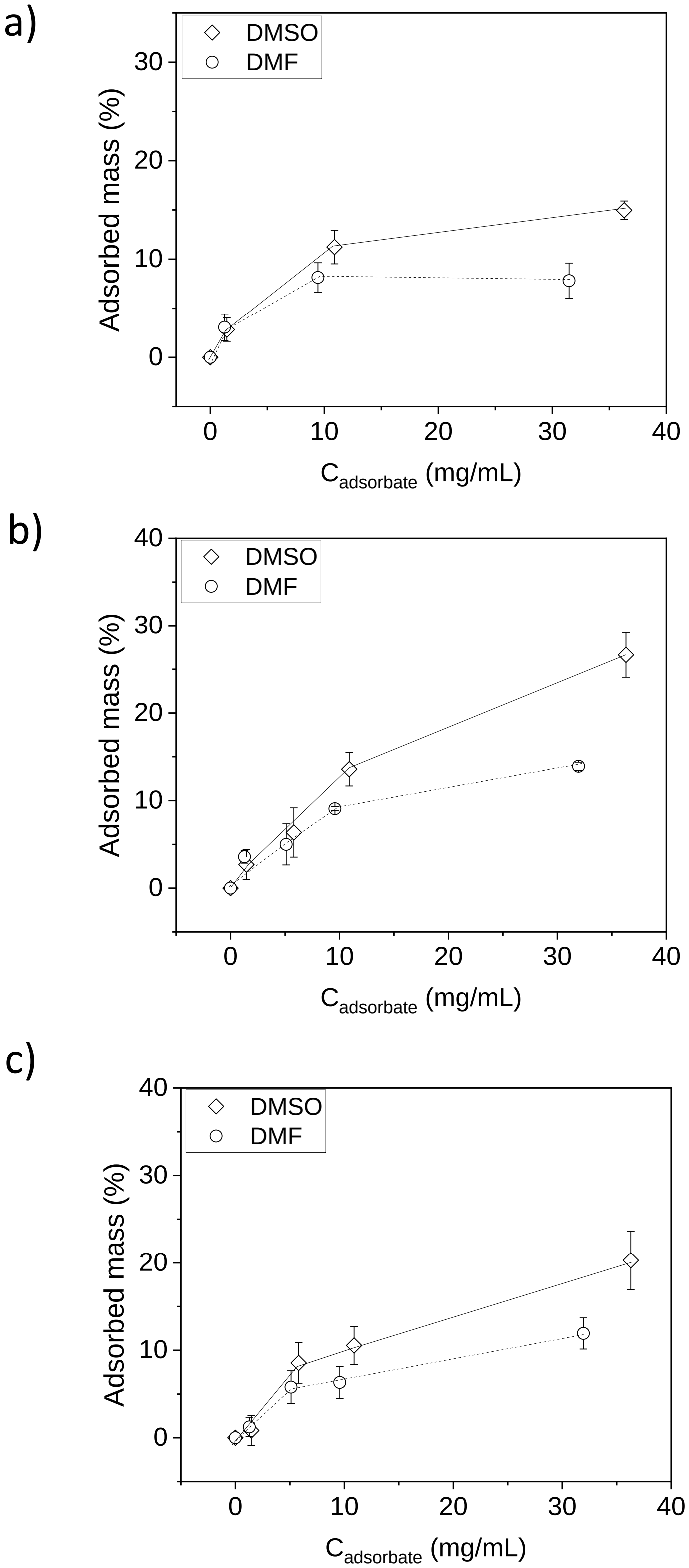

Fig. S5 Adsorption isotherm curves determined by UV-VIS spectroscopy. Adsorption isotherm curves of **a** MIL-88A(Fe), **b** MOF-74(Cu) and **c** MOF-74(Co) measured by ultraviolet spectroscopy for comparative purposes with those in the manuscript. Each measurement was repeated five times in order to provide the corresponding associated statistical error.
