# Supplementary material for: Magnetic sustentation as an adsorption characterization technique for paramagnetic metal-organic frameworks
Source: Commun Chem. 2023 Jan 5;6:4. doi: 10.1038/s42004-022-00799-w (PMC9814357; doi:10.1038/s42004-022-00799-w)
Supplement: Supplementary file 3 — Description of Additional Supplementary Files [file 42004_2022_799_MOESM3_ESM.pdf]

Description of Additional Supplementary Files

File name: Supplementary Movie 1

Description: Experimental procedure to perform the magnetic sustentation experiment
